# Treatment of hypothyroidism with levothyroxine plus liothyronine: a randomized, double-blind, crossover study

**DOI:** 10.1590/2359-3997000000192

**Published:** 2016-08-23

**Authors:** Juliana Kaminski, Fabíola Yukiko Miasaki, Gilberto Paz-Filho, Hans Graf, Gisah Amaral de Carvalho

**Affiliations:** 1 Departamento de Medicina Interna Hospital das Clínicas Universidade Federal do Paraná Curitiba PR Brasil Serviço de Endocrinologia e Metabologia (SEMPR), Departamento de Medicina Interna, Hospital das Clínicas da Universidade Federal do Paraná (HC-UFPR), Curitiba, PR, Brasil; 2 Genome Sciences Department The John Curtin School of Medical Research The Australian National University Canberra ACT Australia Genome Sciences Department, The John Curtin School of Medical Research, The Australian National University, Canberra, ACT, Australia

**Keywords:** Clinical trial, combined modality therapy, cross-over studies, hypothyroidism, levothyroxine, triiodothyronine, liothyronine, quality of life, randomized

## Abstract

**Objective:**

To compare the effects of a unique fixed combination levothyroxine/liothyronine (LT4/LT3) therapy in patients with primary hypothyroidism.

**Subjects and methods:**

This is a randomized, double-blind, crossover study. Adults with primary hypothyroidism (n = 32, age 42.6 ± 13.3, 30 females) on stable doses of LT4 for ≥ 6 months (125 or 150 μg/day) were randomized to continue LT4 treatment (G1) or to start LT4/LT3 therapy (75/15 μg/day; G2). After 8 weeks, participants switched treatments for 8 more weeks. Thyroid function, lipid profile, plasma glucose, body weight, electrocardiogram, vital signs, and quality of life (QoL) were evaluated at weeks 0, 8 and 16.

**Results:**

Free T4 levels were significantly lower while on LT4/LT3 (G1: 1.07 ± 0.29 vs. 1.65 ± 0.46; G2: 0.97 ± 0.26 vs. 1.63 ± 0.43 ng/dL; P < 0.001). TSH and T3 levels were not affected by type of therapy. More patients on LT4/LT3 had T3 levels above the upper limit (15% vs. 3%). The combination therapy led to an increase in heart rate, with no significant changes in electrocardiogram or arterial blood pressure. Lipid profile, body weight and QoL remained unchanged.

**Conclusions:**

The combination therapy yielded significantly lower free T4 levels, with no changes in TSH or T3 levels. More patients on LT4/T3 had elevated T3 levels, with no significant alterations in the evaluated outcomes. No clear clinical benefit of the studied formulation could be observed. Future trials need to evaluate different formulations and the impact of the combined therapy in select populations with genetic polymorphisms.

## INTRODUCTION

Levothyroxine sodium (LT4) is the drug of choice for the treatment of patients with hypothyroidism (1-5). The LT4 formulations available have a half-life of six days and provide fairly stable blood levels of thyroxine (T4) after ingestion of an oral daily dose (6). When treating patients with hypothyroidism, normal blood levels of TSH and free T4 (fT4) are achieved in most patients, with improvements in hypothyroid signs and symptoms.

However, approximately 5-10% of patients continue to report symptoms of hypothyroidism, despite their TSH levels being within the normal reference range (7). Mounting evidence suggests that LT4 monotherapy cannot assure a euthyroid state in the blood and in all tissues simultaneously, and that normal serum thyroid-stimulating hormone (TSH) levels in patients receiving LT4 reflect only pituitary euthyroidism (8). This could be attributed to the fact that the peripheral conversion from T4 to triiodothyronine (T3) is not sufficient to restore normal T3 levels. In animal studies, only the combination levothyroxine/liothyronine (the synthetic form of triiodothyronine; LT4/LT3) ensured euthyroidism in all tissues of thyroidectomized rats (9).

In humans, several clinical trials have evaluated whether LT4/LT3 combination therapy was able to reverse overt hypothyroidism and improve symptoms and quality of life (QoL). The results from the first randomized, double-blind, crossover trial were reported in 1970. These showed no positive results regarding patient preference toward LT4/LT3 therapy and revealed a high incidence of hyperthyroid symptoms (possibly due to excessive doses of LT4/LT3) (10). In 1999, Bunevicius and cols. described an increase in well-being, mood and psychometric functionality in patients treated with LT4/LT3 (11), and similar results were replicated by another group (12). Those findings could not be further replicated by other studies, and recent meta-analyses of those trials did not show any evidence supporting a superior effect of combination treatment (13,14). However, in the clinical setting, some patients who complain of hypothyroid symptoms (despite having normal TSH and fT4 levels) have mentioned improvements when empirically treated with LT4/LT3 and preferred that type of therapy (15). Therefore, it is still unclear whether the combination therapy LT4/LT3 is superior to LT4 monotherapy in patients with hypothyroidism.

The aim of the present study was to compare the effects of a unique, fixed combination LT4/LT3 therapy (75 µg of LT4 and 15 µg of LT3, once a day) on thyroid hormone levels, body weight, vital signs, metabolic parameters, and QoL of patients with primary hypothyroidism.

## SUBJECTS AND METHODS

### Study subjects

The study was approved by the Federal University Hospital of Paraná Ethics Committee, and written informed consent was obtained from all study participants, who were recruited from the Clinical Hospital of the Federal University of Paraná, Curitiba, Brazil. Inclusion criteria were participants of any gender, between 15 and 65 years old, with an established diagnosis of primary hypothyroidism, who had received stable doses of LT4 during the previous six months (125 or 150 μg/day). Exclusion criteria were: diabetes mellitus or serious concomitant diseases (such as liver, renal or heart failure); use of drugs or substances that alter the pharmacokinetics and measures of serum TSH and of thyroid hormones; pregnancy; and use of hormonal contraception. Participants with diagnosis of depression were not excluded, provided they had been receiving adequate antidepressant treatment for the previous six months.

### Study design

This was a randomized, double-blind, crossover study. One group of participants was randomized to continue receiving their usual dose of LT4 for 8 weeks, followed by use of combination therapy LT4 plus LT3 for 8 more weeks (G1). The participants included in the second group (G2) were randomized to switch their usual therapeutic regimen to the combination therapy LT4/LT3 for 8 weeks and to go back to their usual LT4 dose for another 8 weeks. Participants received capsules containing either their usual dose of LT4 (125 μg or 150 μg; Euthyrox, Merck S/A, Brazil) or LT4/LT3 (75 μg of LT4, plus 15 μg of LT3; Novothyral, Merck KGaA, Germany). Participants were advised to take their medication once daily, half an hour before breakfast. At the end of each eight-week period, participants received another batch of capsules. Drug dispensing was performed by one unblinded investigator (G.A.C.). This individual did not participate in the assessment of the results. Adherence to the therapy was assessed by direct questioning during follow-up visits and pill counting. Adverse events were evaluated by standard anamnesis and physical examination.

Participants were evaluated at baseline and at the end of each 8-week period. At each visit, venous blood samples were collected from all participants after a 12-hour fast in order to measure serum TSH, total T3, fT4, glucose, total cholesterol, HDL cholesterol and triglycerides.

### Laboratory methods

TSH was determined in duplicates by Immulite^®^ 2000 chemiluminescence TSH third-generation kit (Diagnostic Products Corporation, CA, USA; reference values [RV] 0.4-4.0 mUI/L; sensitivity 0.002 mUI/L; intra-assay coefficient of variation [CV], 3.8-12.5%; and inter-assay CV, 4.6-12.5%). Free T4 was measured in duplicates by Immulite^®^ 2000 chemiluminescence enzyme-linked immunosorbent assay kit (Diagnostic Products Corporation, CA, USA; RV, 0.8-1.9 ng/dL; sensitivity, 0.15 ng/dL; intra-assay CV, 4.4%-7.5%; and inter-assay CV, 4.8%-9.0%). Total T3 was measured in duplicates by Immulite^®^ 2000 chemiluminescence enzyme-linked immunosorbent assay kit (Diagnostic Products Corporation, CA, USA; RV, 82-179 ng/dL; sensitivity, 19 ng/dL; intra-assay CV, 4.4%-12%; and inter-assay CV, 5.3%-15%). Glucose was measured by enzymatic method (Hexokinase II, Bayer, Germany). Serum total cholesterol, HDL cholesterol and triglycerides were measured by enzymatic colorimetric methods (Sera-Pak Cholesterol Fast Color kit, HDL Advia and Sera-Pak Triglyceride Fast Color kits, Bayer, Germany).

Body weight, resting heart rate and arterial blood pressure were measured at baseline and at the end of each treatment period. Resting heart rate and arterial blood pressure were measured in the sitting position. Twelve-lead electrocardiogram (ECG) was obtained at the end of each treatment period.

### Quality of life evaluation

At each visit, QoL was assessed using a disease-specific questionnaire adapted from the Health Related Quality of Life (HRQOL) questionnaire (16), which in turn was elaborated to detect impaired well-being in subjects with hypothyroidism. We selected 29 items from the HRQOL questionnaire and added four items: palpitation, insomnia, irritability and anxiety. Therefore, our questionnaire consisted of 33 items grouped into three categories: physical complaints (12 items), energy and general well-being (11 items), and mood and emotions (10 items). Scoring was based on a six-point scale from zero to five, with zero representing a more favorable state. Participants also completed a visual analog scale (VAS) at baseline, 8 weeks and 16 weeks that assessed overall well-being, interest in sex and ability to engage in physical activities, social interactions and work. In the VAS, scores from -2.5 (the worst possible state) to +2.5 (the best possible state) were given for each of the five questions in 0.25-point increments (overall feeling, ability to engage in physical activity, ability to engage in social interaction, ability to work and interest in sex). Each patient was evaluated by the same blinded examiner during all three sessions (either J.K. or F.Y.M.).

### Statistical analysis

Clinical and biochemical data were analyzed by one-way ANOVA, Dunn’s multiple comparison test, paired or unpaired (where appropriate) t-test for variables with normal distribution, and Wilcoxon signed-rank test or Mann-Whitney U test for variables without normal distribution. To compare sample proportions, a z-test was employed. Quality of life scores were analyzed by Friedman rank-sums test. Correlations between QoL scores and thyroid hormone levels were calculated using Spearman’s or Pearson’s correlation coefficients. All variables were tested for normality by Kolmogorov-Smirnov test. Analyses were performed using GraphPad Prism 5.04.

## RESULTS

**Figure 1:**
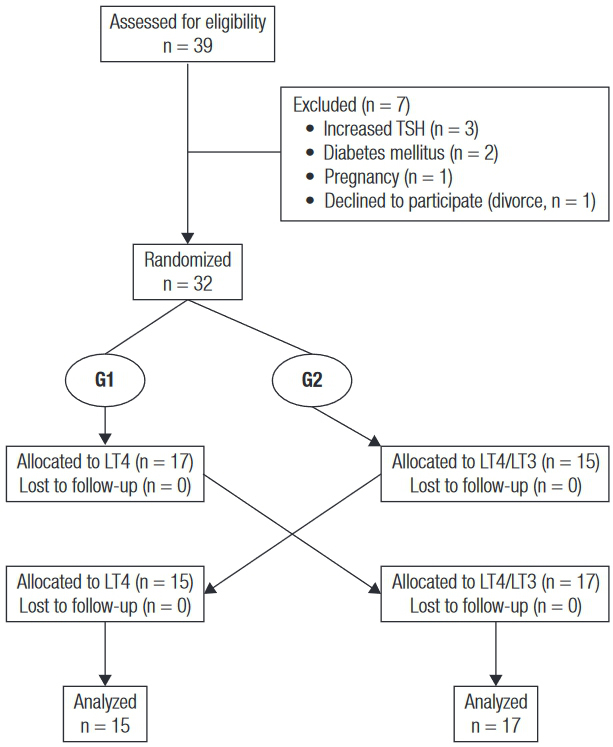
CONSORT diagram.

A total of 39 patients were considered for inclusion. Seven patients were excluded before randomization, one because of pregnancy, one because of divorce, two because of onset of diabetes mellitus, and three because of increased TSH levels during the previous six months (Figure 1).

Thirty-two participants who fulfilled the inclusion and exclusion criteria were recruited. Twenty-three participants (71.9%) had autoimmune or idiopathic hypothyroidism, six (18.7%) had post-surgical hypothyroidism acquired after therapy for differentiated thyroid carcinoma, and three (9.4%) had radioiodine-induced hypothyroidism due to Graves’ disease. Mean age was 42.6 ± 13.3 years old, and 94% were female. The median duration of hypothyroidism was 4.5 years (minimum 1, maximum 33 years). The same proportion of participants was being treated with either 125 μg or 150 μg of LT4 per day. Participants were randomized to continue taking their usual LT4 dose (G1; n = 17) or to switch to the LT4/LT3 combination therapy (G2; n = 15). Baseline clinical and biochemical data were similar for both groups (Table 1). Serum basal TSH levels ranged from 0.001 to 4.5 mU/L in G1 and from 0.001 to 8.425 mU/L in G2. Six patients had suppressed TSH as a result of treatment for thyroid carcinoma. On the contrary, some patients had increased TSH levels at the time of randomization. Ideally, serum basal TSH concentrations would be within normal range. However, we decided to keep all patients in the statistical analysis because each crossover patient served as his or her own control.

**Table 1:** Baseline clinical and biochemical data

	Prior to randomization (n = 32)	Group 1 (G1) (n = 17)	Group 2 (G2) (n = 15)	P (group 1 vs. group 2)
Age (years-old)	42.6 ± 13.3	39.5 ± 14.2	46.1 ± 11.86	0.170
Gender (F:M)	30:2	15:2	15:0	0.143
Participants on LT4 125 μg/day (%)	50	52.9	46.7	0.726
Time of hypothyroidism diagnosis (years)	4.5 (1 – 33)	5 (1 – 33)	4 (1 – 23)	0.595
Autoimmune/idiopathic hypothyroidism (%)	71.9	58.8	86.7	0.080
Post-surgical hypothyroidism (%)	18.7	23.5	13.3	0.460
Radioiodine-induced hypothyroidism (%)	9.4	17.6	0	0.088
Body mass index (kg/m^2^)	28.5 ± 5.6	27.3 ± 5.0	29.8 ± 6.0	0.211
Resting heart rate (beats per minute)	76.7 ± 9.8	79.1 ± 9.0	74.1 ± 10.3	0.160
Systolic blood pressure (mmHg)	120.0 (90.0 – 150.0)	120.0 (100.0 – 140.0)	120.0 (90.0 – 150.0)	0.612
Diastolic blood pressure (mmHg)	78.9 ± 11.3	77.1 ± 11.2	81.0 ± 11.5	0.335
TSH (mU/L)	0.309 (0.001 – 8.425)	0.078 (0.001 – 4.500)	0.535 (0.001 – 8.425)	0.117
Total T3 (ng/dL)	93.6 ± 11.6	93.2 ± 11.6	94.0 ± 12.0	0.847
Free T4 (ng/dL)	1.26 ± 0.26	1.26 ± 0.25	1.26 ± 0.28	0.956
Glucose (mg/dL)	99.0 ± 12.5	97.6 ± 10.6	100.5 ± 14.5	0.532
Total cholesterol (mg/dL)	191.1 ± 35.4	188.8 ± 36.9	193.7 ± 34.8	0.706
HDL (mg/dL)	47.2 ± 11.9	47.5 ± 12.4	46.9 ± 11.8	0.891
Triglycerides (mg/dL)	139.7 ± 68.0	144.5 ± 75.0	134.3 ± 61.2	0.679

Note: results are shown as mean ± standard deviation or median (minimum – maximum).

**Figure 2:**
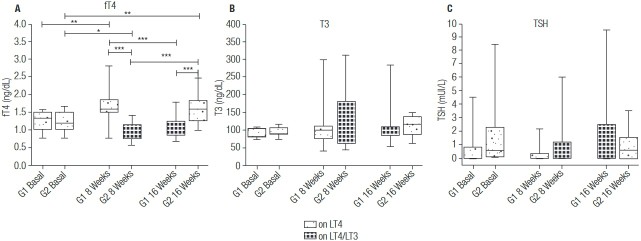
Changes in thyroid function tests.

Over the 16-week period, significant changes were observed only for fT4 levels (one-way ANOVA P < 0.001 in both groups), with significantly lower levels observed while taking LT4/LT3 treatment (1.07 ± 0.29 at week 16 vs. 1.65 ± 0.46 ng/dL at week 8 in G1; 0.97 ± 0.26 at week 8 vs. 1.63 ± 0.43 ng/dL at week 16 in G2) (Figure 2A). T3 levels were similar over the study in G1 (one-way ANOVA P = 0.662) and G2 (one-way ANOVA P = 0.247), and median T3 levels were not affected by type of therapy (Figure 2B). Changes in TSH levels were not significant in G1 (one-way ANOVA P = 0.05) or in G2 (one-way ANOVA P = 0.819; Figure 2C).

Only statistically significant comparisons are depicted.

* P < 0.05; ** P < 0.01; *** P < 0.001.

G1: Group 1, on LT4 monotherapy; G2: Group 2, on LT4/LT3 combination therapy.

The analysis of the results according to type of therapy (LT4 or LT4/LT3) showed that fT4 levels were significantly lower and resting heart rate was slightly higher while participants were taking the combination therapy. Other outcomes were similar, regardless of type of therapy (Table 2). Furthermore, five patients had T3 > 180 ng/dL while on LT4/LT3 (mean 239.4 ± 57.1 ng/dL, highest 311.5 ng/dL), whereas only one participant had increased T3 levels (298.5 ng/dL) when on LT4 therapy.

**Table 2:** Clinical data, thyroid function tests and biochemical results while on LT4 or LT4/LT3

	On LT4 monotherapy	On LT4/LT3 combination therapy	P
Body mass index (kg/m^2^)	28.5 ± 5.7	28.4 ± 5.7	0.212
Resting heart rate	74.6 ± 9.5	77.4 ± 9.2	0.046
Systolic blood pressure (mmHg)	118.1 ± 13.3	117.5 ± 11.1	0.875
Diastolic blood pressure (mmHg)	75.6 ± 9.0	74.1 ± 8.7	0.446
TSH (mU/L)	0.189 (0.004 – 3.495)	0.638 (0.013 – 9.500)	0.540
T3 (ng/dL)	103.8 (40.0 – 298.5)	98.5 (44.7 – 311.5)	0.742
Free T4 (ng/dL)	1.64 ± 0.44	1.03 ± 0.28	< 0.0001
Glucose (mg/dL)	102.9 ± 23.7	99.0 ± 14.1	0.191
Total cholesterol (mg/dL)	188.4 ± 31.3	190.9 ± 34.9	0.493
HDL (mg/dL)	49.1 ± 10.1	48.2 ± 10.4	0.286
Triglycerides (mg/dL)	130.6 ± 53.4	135.7 ± 66.6	0.623

Note: results are shown as mean ± standard deviation or median (minimum – maximum).

No significant adverse effects were reported, and all subjects completed the study. Although resting heart rate was slightly higher during the combination LT4/LT3 treatment, it remained within the normal range. In addition, no arrhythmias were detected on resting ECG.

**Figure 3:**
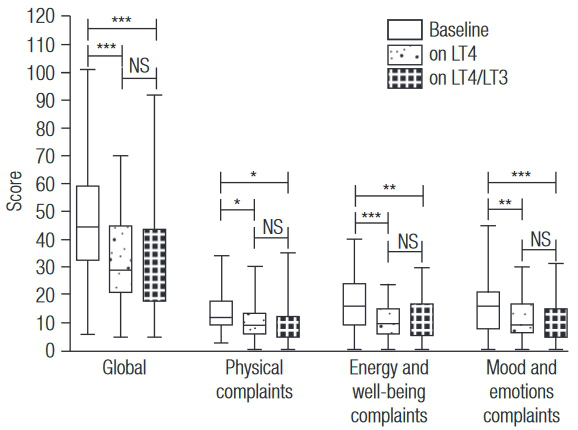
Scores of quality of life, global and by type of complaint.

All participants responded to the adapted HRQOL questionnaire. Global median scores improved after randomization (P < 0.001). Scores categorized into subgroups (physical complaints, energy/general well-being, and mood/emotions) also improved after randomization (P < 0.05). However, there was no difference in global scores when comparing the two types of therapy (P = 0.888). Furthermore, scores in each subgroup were similar for both treatments (all P > 0.05) (Figure 3). There were no correlations between global scores and thyroid hormone levels (fT4 and T3) at baseline or while on LT4 or LT4/LT3 (all P > 0.05). However, there was a positive correlation between TSH and global scores while on LT4/LT3 (r = 0.4164, P = 0.018). Correlation analysis between scores by subgroups and thyroid hormone levels showed correlation only between energy/general well-being scores and TSH levels while on LT4/LT3 (r = 0.4123, P = 0.019) and between mood/emotions scores and TSH levels, also while on LT4/LT3 (r = 0.4011, P = 0.023).

Box-plots depict median and range.

* P < 0.05; ** P < 0.01; *** P < 0.001.

By analyzing the 33 items of the adapted HRQOL questionnaire individually, we observed that 15 items had significantly different median scores when comparing scores at baseline to scores when on LT4 to scores of LT4/LT3 combination therapy (P < 0.05). According to these scores, QoL improved for all 15 of these specific items. When analyzing the effect of each type of therapy within these 15 items, lack of improvement from baseline was observed more frequently when participants were on LT4/LT3 combination therapy. Median scores did not change for 3 items while participants were on LT4/LT3, whereas scores remained the same for only one item while participants were on LT4. Conversely, compared to baseline, 14 items had their scores improved while on LT4; on LT4/LT3, lower scores were obtained in 12 items of the adapted HRQOL questionnaire. In a *post hoc* analysis, only two items were significantly different when the two treatments were compared. Median scores concerning brittle nails were lower while on LT4, and scores regarding shortness of breath were lower while on LT4/LT3. Concerning the four items that were added to the original questionnaire (palpitation, insomnia, irritability and anxiety), no significant changes were observed between the two regimens (Table 3).

**Table 3:** Itemized scores of quality of life

N	Symptom	Prior to randomization	On LT4	On LT4/LT3	P value (baseline vs. LT4 vs. LT4/LT3)	P value (baseline vs. LT4) *Post hoc*	P value (baseline vs. LT4/LT3) *Post hoc*	P value (LT4 vs. LT4/LT3) *Post hoc*
**Physical complaints**	Feeling cold	0 (0-4)	0 (0-2)	0 (0-4)	0.032	0.018	0.025	0.904
	Dry skin	1.5 (0-5)	1.5 (0-4)	1.5 (0-4)	0.007	0.002	0.031	0.275
	Cold skin	0 (0-4)	0 (0-5)	0 (0-3)	0.640	NA	NA	NA
	Tightening of clothes	1 (0-5)	0 (0-5)	0 (0-5)	0.113	NA	NA	NA
	Dry hair	0.5 (0-5)	0.5 (0-5)	0 (0-5)	0.076	NA	NA	NA
	Puffiness of hands	1 (0-3)	0 (0-3)	0 (0-3)	0.013	0.006	0.013	0.778
	Brittle nails	0 (0-5)	0 (0-3)	1 (0-5)	0.013	0.021	0.557	0.004
	Muscle cramps	0.5 (0-5)	0 (0-5)	0 (0-5)	0.182	NA	NA	NA
	Shortness of breath	1 (0-4)	1.5 (0-3)	1 (0-5)	0.018	0.453	0.006	0.038
	Swollen feet	1 (0-5)	0 (0-4)	0 (0-3)	0.071	NA	NA	NA
	Pins and needles in hands/feet	0 (0-3)	0 (0-3)	0 (0-5)	0.433	NA	NA	NA
	Palpitation	1 (0-5)	1 (0-5)	0.5 (0-5)	0.182	NA	NA	NA

**Energy and general well-being**	Feeling tired	2.5 (0-5)	2 (0-4)	1 (0-5)	0.002	0.003	0.002	0.879
	Feeling need for more sleep	1.5 (0-5)	1 (0-3)	1 (0-4)	0.587	NA	NA	NA
	Needing nap during day	1 (0-5)	1 (0-3)	1 (0-3)	0.220	NA	NA	NA
	Needing more time to do daily chores	1 (0-5)	0 (0-4)	0 (0-3)	0.001	0.003	0.001	0.758
	Insomnia	0 (0-5)	0 (0-5)	0 (0-5)	0.658	NA	NA	NA
	Slower physically	1 (0-5)	0 (0-3)	0.5 (0-3)	0.013	0.012	0.006	0.797
	Slower mentally	2 (0-5)	1 (0-4)	1 (0-5)	< 0.001	0.002	< 0.001	0.340
	Needing more time for calculations	1.5 (0-5)	1 (0-5)	1 (0-4)	0.044	0.013	0.128	0.307
	Going out less	1 (0-5)	1 (0-4)	1 (0-5)	0.120	NA	NA	NA
	No energy to get through the day	2 (0-5)	1 (0-5)	1 (0-4)	0.001	< 0.001	0.016	0.135
	Slowing of movements	0 (0-5)	0 (0-2)	0 (0-5)	0.072	NA	NA	NA

**Mood and emotions**	Feeling frustrated	0 (0-5)	0 (0-4)	0 (0 -3)	0.005	< 0.001	0.016	0.135
	Feeling discouraged	2 (0-5)	1 (0-5)	1 (0-5)	0.002	0.001	0.004	0.538
	Difficulty concentrating	1 (0-5 )	1 (0-4)	1 (0-4)	0.093	NA	NA	NA
	Deterioration of memory	1 (0-5)	1 (0-4)	1 (0-4)	0.066	NA	NA	NA
	Losing interest in sex	1.5 (0-5)	1 (0-5)	1 (0-5)	0.476	NA	NA	NA
	Losing interest in activities/hobbies	1 (0-5)	1 (0-3)	1 (0-4)	0.495	NA	NA	NA
	Feeling depressed	1 (0-5)	0 (0-5)	0 (0-5)	0.021	0.005	0.118	0.191
	Feeling worthless	0 (0-5)	0 (0-2)	0 (0-3)	0.971	NA	NA	NA
	Irritability	2 (0-5)	1 (0-5)	1 (0-5)	0.011	0.033	0.003	0.359
	Anxiety	2 (0-5)	1 (0-5)	1 (0-5)	0.069	NA	NA	NA

Note: scores are shown as median (minimum – maximum). Scoring was based on a six-point scale from zero to five, with zero representing a more favorable state.

NA: not applicable; G1: Group 1, on LT4 monotherapy; G2: Group 2, on LT4/LT3 combination therapy.

Mean VAS scores were similar at baseline (0.47 ± 0.91), while on LT4 (0.21 ± 0.91), and while on LT4/LT3 (0.44 ± 1.26; one-way ANOVA P = 0.5175).

We performed a sub-analysis according the etiologies of hypothyroidism: autoimmune or idiopathic hypothyroidism, post-surgical hypothyroidism acquired after therapy for differentiated thyroid carcinoma and radioiodine-induced hypothyroidism due to Graves’ disease. No significant changes were observed in VAS scores or QoL scores (global and subgroup scores) between the two treatments. We also performed a subanalysis for the group with high T3 levels (T3 > 180 ng/dL), which showed no significant differences regarding QoL scores (global and subgroup scores), VAS scores, body mass index, arterial blood pressure or resting heart rate.

## DISCUSSION

Currently, the drug of choice for the treatment of hypothyroidism is levothyroxine sodium (1-5), and LT4/LT3 combination therapy should be considered solely as an experimental treatment modality (7). There is conflicting animal and human data suggesting that the addition of liothyronine to levothyroxine therapy improves outcomes (15). To clarify the role of liothyronine in the treatment of hypothyroidism, we conducted a randomized, double-blind, crossover clinical trial in which participants received LT4 alone for 8 weeks and LT4/LT3 for another 8 weeks. Our results suggest that the combination therapy LT4/LT3 significantly determines lower serum free T4 levels without significantly altering serum TSH, total T3, lipids, or fasting glucose levels. The type of treatment implemented did not affect body mass index and arterial blood pressure. Despite the fact that resting heart rate was slightly higher in participants taking the combination therapy, neither ECG alterations nor significant adverse effects were observed. Furthermore, no differences were observed regarding overall quality of life, and LT4/LT3 therapy was significantly superior to LT4 for only one of the 33 items evaluating quality of life.

Autoimmune thyroid disease is present in about 70% of hypothyroid individuals, and patients with autoimmune thyroid disease are at high risk of developing other autoimmune diseases. The development of these associated conditions might go unnoticed, and some patients have persistent nonspecific symptoms despite apparently adequate levothyroxine. In addition, there is the possibility that thyroid autoimmunity itself might give rise to nonspecific symptoms, independent of thyroid function (8).

The adult thyroid gland secretes all the circulating T4 and 20% of T3 present in the blood. The remaining 80% of the circulating T3 derives from peripheral 5’-deiodination of the secreted T4 (17). Evidence from thyroidectomized animal and human studies suggests that thyroid hormone replacement therapy in the form of LT4 is not sufficient to lead to euthyroidism or to replenish 100% of the circulating and tissular T3, even assuming that peripheral conversion from T4 to T3 is normal (18,19). In fact, a true euthyroid state (in plasma and tissue) may be attained only when LT4/LT3 combination therapy is employed (9). This piece of evidence, along with the observation that many hypothyroid patients do not have their symptoms improved with LT4 therapy alone despite normal thyroid function tests, led to the development of human trials assessing the efficacy of LT4/LT3 in the treatment of hypothyroidism.

To date, 15 clinical trials have evaluated the combination therapy of LT4 plus synthetic liothyronine in patients with hypothyroidism [reviewed by Escobar-Morreale and cols. (15)], and one study evaluated desiccated thyroid extract containing both T3 and T4 (20). Clear benefits of the combination therapy were only observed among two groups from Lithuania and Denmark, which reported improvements in mood, well-being and psychometric functionality (11,21), along with better scores of QoL, depression and anxiety (12). In the first trial showing positive results, the significant benefit of the combination therapy was evident only in a group of athyreotic subjects who had previously had thyroid cancer. Thus, it is not clear whether the results were related to the cause of hypothyroidism (21). In the other positive study, treatment dose was titrated to aim for stable serum TSH levels. Significant improvements in QoL, depression and anxiety scales were observed with a considerably lower T4:T3 ratio of 2.5:1 (12). The study with the largest sample size and longest follow-up was conducted by Saravanan and cols. In that study, 697 patients with primary hypothyroidism were initially randomized and treated in a non-crossover, double-blind design at a T4:T3 weight ratio of 5:1. That study reported a slight improvement in mood, QoL and anxiety after 3 months of the combination treatment, which was not confirmed subsequently after 1 year (22). Furthermore, a meta-analysis evaluating 11 randomized controlled trials with a total of 1,216 patients concluded that there is no evidence supporting the superiority of LT4/LT3 combination therapy regarding improvements in bodily pain, mood, fatigue, QoL, cognition, body weight or blood lipids (13). Not even patients with depression (11), high dissatisfaction with LT4 therapy (23), psychiatric symptoms (24), or fatigue (25) benefited from the combination therapy.

In our study, we evaluated 32 participants with stable, treated primary hypothyroidism, most of them of autoimmune or idiopathic etiology. However, some patients were athyreotic following treatment for thyroid cancer or had hypothyroidism because of the administration of radioactive iodine therapy for Graves’ disease. Also, both genders were included in the study; therefore, this heterogeneity may be a confounding factor, as previously observed (21). Instead of using a substitution approach to calculate the dose of LT4/LT3, we recruited participants who were within a very limited LT4 daily dose range (125 or 150 μg/day) and used a fixed combination approach. We administered LT4/LT3 at a fixed dose of 75 μg of LT4 plus 15 μg of LT3, regardless of their initial LT4 dose, in the same 5:1 weight ratio as previously evaluated in other studies (12,22-24). Doses were not adjusted during the study period, as was done by Nygaard and cols. (12). To our knowledge, no other studies have employed such strict recruitment criteria, and the fixed dose of 75 µg of LT4 plus 15 µg of T3 has been evaluated only by one study (24). That study recruited participants taking any dose of LT4, and a substitution approach was employed to calculate the LT4/LT3 dose for a subgroup of patients (usual LT4 dose minus 25 µg of LT4 plus 15 µg of LT3). Within this subgroup of patients that was probably overtreated, half of the participants underwent TSH suppression and experienced increases in heart rate and significant weight loss. Nygaard and cols.’s study recruited patients on different daily doses of LT4; a few participants whose dose prior to randomization was 125 µg/day were treated with a dose that was close but not equal to ours (75 µg of LT4 + 20 µg of T3). In the study by Clyde and cols., a few participants who were initially on LT4 125 µg/day received the same combination dose as the one employed in our study. However, the LT3 dose was administered twice daily (26).

We submitted participants to both types of therapy for 8 weeks each, which led to significantly lower plasma levels of fT4 while on LT4/LT3 therapy. This is an expected finding that was reported in a previous meta-analysis, which also reported no effect of the combination therapy on plasma TSH, total T3 or lipids (13). In our study, comparisons of results observed at baseline to those observed at 8 weeks and at 16 weeks also showed that fT4 levels were lower when participants were under LT4/LT3 therapy. Also, there were no changes in TSH and T3 levels over the 16-week period. Although we could not identify increases in T3 levels caused by the combination therapy, it is possible that these results are falsely low due to the short half-life of liothyronine, as well as the fact that blood samples were collected 24 hours after administration of the LT4/LT3 tablets.

Although the T4:T3 ratio of 5:1 used in our study is similar to those used in other studies [but different than the 13:1-20:1 weight ratio recommended by the European Thyroid Association (7)], we have not assessed whether that dose led to both blood and tissular euthyroidism. Excessive doses of LT4/LT3 may contribute to increases in resting heart rate (27), bone remodeling markers (24,28-30) and serum aminotransferases (30), leading to clinical manifestations of hyperthyroidism (31). In our study, we observed a borderline significant increase in heart rate by 3 beats/minute, which did not lead to adverse events. Even though atrial arrhythmias have been reported in two studies in which patients were overtreated during the combination treatment (10,32), we did not detect arrhythmias on resting 12-lead ECG. Furthermore, no significant changes were detected in arterial blood pressure, in concordance with previous clinical trials (10-12,22-24,32).

We also evaluated body weight (as this can be affected by slight variations in thyroid hormone levels), body composition and resting energy expenditure (33). In one previous study, significant decreases in body weight after the combination therapy were observed. However, the combination therapy led to overtreatment in many patients, obtaining a median TSH of 0.07 mU/L (24). Nevertheless, similar to our results, other clinical trials did not reveal significant changes in body weight after LT4/LT3 therapy (11,12,22,23).

Overt hypothyroidism is classically associated with negative effects on lipid metabolism (34), which improves significantly with LT4 therapy (35,36). However, very few trials showed a benefit of the LT4/LT3 combination treatment on blood lipids (37). In our study, we did not observe significant changes in total cholesterol, LDL-cholesterol, HDL-cholesterol or triglycerides, in concordance with previous studies (11,22). Additionally, the combination therapy did not affect fasting glucose levels. Since we did not measure fasting insulin levels, we could not assess the impact of LT4/LT3 therapy on insulin sensitivity.

Hypothyroidism may induce affective and cognitive dysfunction (38,39). Most studies have assessed changes in mood, cognition function and QoL during combination therapy with LT4/LT3 (11,12,21,23-26,28,30-32,40,41). Two meta-analyses including 10 (14) and 11 (13) of these studies showed no improvement in bodily pain, well-being, mood, fatigue, QoL or cognition. It should be noted that the evaluation of QoL, well-being, mood and thyroid symptoms is not standardized across studies, and different instruments (such as the SF-36, Hamilton Scales of Depression and Anxiety, and Thyroid Symptom Questionnaire) have been employed. In our study, we adapted and translated to Portuguese the questionnaire developed and employed by Jaeschke and cols. (16) and Clyde and cols. in their clinical trials (26). The analysis of our adapted HRQOL questionnaire revealed no significant changes in global scores or in scores within each category (physical complaints, energy and well-being, and mood and emotions). Interestingly, in 15 items of the questionnaire, we observed improvement in the scores throughout the study period when compared with baseline scores. This could be explained by the Hawthorne effect, or the tendency of patients to describe improvement in health simply due to participation in a trial (6). However, when excluding the baseline scores and performing the *post hoc* analysis between the two treatments, only two out of the 33 evaluated items had significantly different scores. Participants on LT4 reported improvement in the “brittle nails” complaint, whereas those on LT4/LT3 reported reduced shortness of breath. In hypothyroid patients, breathing complaints are unspecific and their clinical importance is unclear. By contrast, fragile nails are the most prevalent symptom in patients with nail involvement in hypothyroidism (42). Nevertheless, it is unclear why this symptom was improved by LT4 therapy. When correlating scores with TSH, fT4 and T3 levels, we could identify only moderate correlation between TSH and global scores, between TSH and energy/general well-being scores, and between TSH levels and mood/emotions scores (all while on LT4/LT3). The lack of effect on QoL was also evident in the analysis of the scores obtained from the VAS.

Whereas a previous study showed that LT4 monotherapy leads to no change in serum free T3 in the first 4 hours post-dose, LT4/LT3 therapy can determine a marked rise by 42% in serum free T3 within the first 4 hours after ingestion of LT4/LT3 (27). This acute rise can cause hyperthyroid symptoms that were not detected by our adapted HRQOL questionnaire. We added four items to that questionnaire (palpitation, insomnia, anxiety and irritability) because we postulated that LT4/LT3 therapy could induce some complaints that would have been missed by the original HRQOL questionnaire. Among those four items, only irritability scores improved from baseline with both therapies. However, those scores were not significantly different from each other.

We performed a 16-week crossover trial to eliminate any carryover effect. By doing so, we observed that TSH levels while on LT4 or LT4/LT3 were similar, and TSH levels remained fairly constant throughout the study. Moreover, we used a commercially available presentation of LT4/LT3 that can be applied in clinics, thereby avoiding dosing errors when compounding liothyronine. However, we did not assess patient preference, which might be higher when taking LT4/LT3 and unrelated to improvements (or lack thereof) in quantitative scores (20). Furthermore, we did not employ a washout period between the LT4 treatment and the combined therapy, to avoid submitting our participants to unnecessary clinical hypothyroidism. In addition, oral liothyronine has a shorter half-life, and significant results could have been observed if we instead had administered a constant, steady supply of T3 by means of enteric, transdermal or intramuscular sustained-release preparation or twice daily (8). Different results could have been observed in a more homogeneous, larger sample, but several similar trials evaluated ≤ 40 participants, with adequate statistical power (11,21,25,28,30,32,37,41). Similarly, the duration of our study may not have been long enough to detect changes in peripheral parameters. However, combination therapy has been evaluated for ≤ 8 weeks in other similar trials (10,11,21,28). Finally, despite being validated in English, our HRQOL questionnaire did not undergo validation in Portuguese.

Despite the fact that our study and previous meta-analyses failed to find clear benefits in the treatment of hypothyroid individuals with combination LT4/LT3, it is possible that a subgroup of patients with deiodinase 2 (*DIO2*) polymorphisms can benefit from combination therapy (43). One such polymorphism in the D2 gene (Th92Ala) is associated with reduced T4 to T3 activation in skeletal muscle and thyroid, as well as alterations in thyroid–pituitary feedback. A suggestive indication to the presence of this polymorphism could be a higher-than-normal ratio of free T4 to free T3 (6,44). Other polymorphisms in the phosphodiesterase 8B (*PDE8B)* may also alter the carrier’s genetically determined TSH set-point, leading to sustained hypothyroid symptoms despite normal TSH levels (7). To date, it is still unclear whether polymorphisms play a role in response to therapy in patients with hypothyroidism (45-47).

Even though animal studies support combined levothyroxine plus liothyronine therapy, clinical trials in humans have not shown the advantages of that approach over administration of levothyroxine alone. The preference of some patients for combined therapy (found in some trials) may have a genetic background. Hence, this should be balanced against the possibility of adverse events resulting from the addition of liothyronine to levothyroxine. Currently available oral liothyronine preparations have an inadequate pharmacokinetic profile. Similarly, commercial preparations that contain levothyroxine and liothyronine contain an excess of the latter and do not mimic the proportion of levothyroxine to triiodothyronine present in normal human thyroidal secretion. Future studies need to evaluate the effect of long-acting, slow-release forms of T3, which mimic normal physiological endogenous T3 production. Furthermore, prospective and longer trials are required to further evaluate the response to the combination LT4/LT3 treatment in patients with the polymorphisms in genes affecting thyroid economy.
